# Use of Social Media for Patient Education in Dermatology: Narrative Review

**DOI:** 10.2196/42609

**Published:** 2023-04-14

**Authors:** Magda Sara Wojtara

**Affiliations:** 1 Department of Human Genetics University of Michigan Medical School Ann Arbor, MI United States

**Keywords:** dermatology, health literacy, innovation, patient education, social media

## Abstract

**Background:**

Social media has rapidly become one of the main avenues for news and communication among those with access to technology. Nearly 60% or 4.7 billion people worldwide use social media. Different social media networks provide users with a barrage of posts, opinions, and transformations. With this noticeable uptick in physician and patient education usage of social media, exploration of the impacts of social media on patient education in dermatology is crucial.

**Objective:**

The goal of this narrative review was to evaluate existing peer-reviewed literature examining the use of social media for patient education in dermatology and to establish trends and implications. Additional attention was given to different social media sites, and potential differences in modalities of posts such as short-form videos on TikTok and Instagram Reels, long-form videos on YouTube, and infographics on Twitter, Instagram, and Facebook.

**Methods:**

PubMed, Access DermatologyDxRx, and Scopus searches of peer-reviewed publications were performed to discover articles with social media and patient education keywords in combination with other health care–relevant or dermatology-relevant keywords. Subsequently, the screening of these studies was performed by the author who has experience with education and research experience in health care, dermatology, social media, and telehealth. Ultimately, the selected articles were summarized through qualitative analysis of key points and presented for further discussion.

**Results:**

Through this narrative review, the researcher was able to identify several publications focusing on dermatology and social media. Some common subject areas included the use of social media for the promotion of private dermatology practices, residency programs, and research journals. So long as providers, such as dermatologists, take ethical considerations into account, these platforms can provide patients with curated educational content. In addition, several publications emphasized the use of social media as a form of patient education on dermatologic conditions but also as a source of misinformation.

**Conclusions:**

This narrative review illuminated the use of social media as a form of patient education for dermatology, with its applications addressed across many demographics and situations. As social media platforms continue to update their algorithms, content filters, and posts, social media may become a reputable form of patient education in dermatology. Future studies and innovations should continue to explore innovations in this space, the efficacy of different modalities of posts, and longitudinal differences in patient outcomes and health literacy.

## Introduction

The popularity of social media platforms has increased over the past decade with nearly 60% or 4.7 billion people worldwide using social media [1]. These platforms allow users to engage with people beyond their in-person social sphere, and many users use social media for anything from catching up on news to learning about a topic. Different social media platforms allow for different user experiences to cater to their different wants and needs. Considering the COVID-19 pandemic, even more time has been spent on social media and on the internet. The average daily time (in minutes spent on social media worldwide) has increased to 63.3% from 2012 to 2022 [2]. Over time, social media usage has become a mainstay in the daily routine of billions.

Among social media’s many uses, a few are of crucial and rising importance: a source of misinformation, a source of patient education, and a promotion for private practices and academic research. Of all dermatology content created on social media platforms, content creators consisted of nonphysicians (52%), physicians (32%) of which 84% were dermatologists, and private companies (16%) [3]. This suggests that much of the content currently circulating is not created by dermatologists. Many platforms recognize that content requires additional moderation to combat the spread of misinformation. For example, Facebook and Instagram have recently instituted third-party fact-checking software, and TikTok has a duet feature that allows physicians to dispel misinformation directly [4]. Health misinformation can lead to a plethora of issues. Some examples include undergoing risky procedures or taking remedies without a clear idea of the potential benefits and side effects. Some patients are now using social media to discover and determine which dermatology practices to frequent. Indeed, in a recent survey, 66% of participants indicated that they went to a dermatologist that they knew about and 21% of participants indicated that they knew about their dermatologist solely from social media content alone [5]. It is likely that as the number of dermatologists using social media for their practices increases, this number may increase as well.

As social media platforms continue to expand and develop new features, understanding how to tackle misinformation and use social media for patient education in dermatology are crucial areas for consideration. Given social media’s increasing popularity as a source of information and their established utility as a means of enhancing human communication, research must consider their growing influence on patients and the spread of current innovations. Effective social media practices are crucial to building a community of users that trust the information that they are being presented with. As such, physicians should be aware of and develop effective social media practices to cater to younger populations without sacrificing patient privacy or professionalism. Integrating social media as a component of private practices, programs and academic research journals can help combat misinformation, promote health literacy, and allow patients to make more informed decisions about their care [6]. Furthermore, if used properly, social media can also help to increase diversity and inclusivity by increasing the opportunity for more open discussions among trainees, physicians, and patients, with different skin conditions across a variety of demographics [7].

## Methods

**Figure 1:**
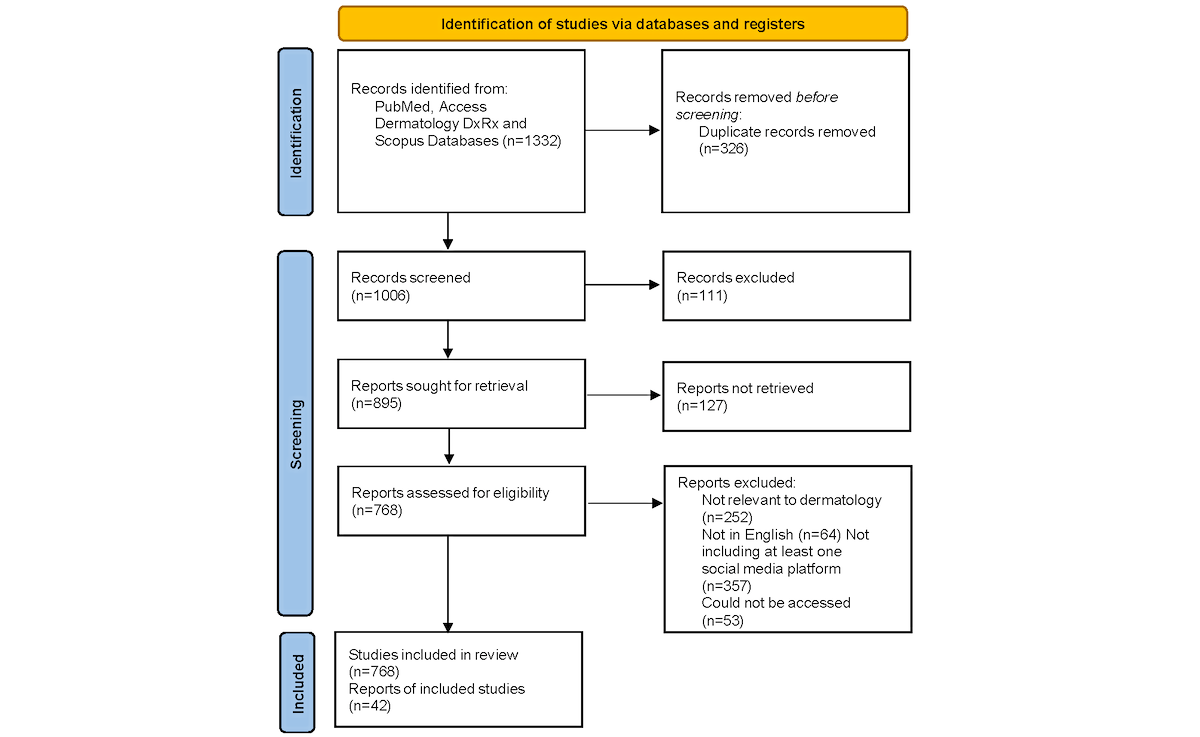
PRISMA (Preferred Reporting Items for Systematic Reviews and Meta-Analyses) 2020 flow diagram for the identification of studies [8].

A PubMed, Access DermatologyDxRx, and Scopus survey of peer-reviewed publications was conducted from July 2022 to January 2023 to discover relevant articles related to social media and social media usage in the contexts of dermatology and academia. The publications selected were published between May 2014 and January 2023. PubMed, Access DermatologyDxRx, and Scopus databases were used for this narrative review although many duplicate sources were found. It is important to note that many of the articles had a specific focus on 1 or 2 social media sites; however, some articles drew broader comparisons across all social media platforms. For clarity, we broadly discuss social media platforms and emphasize specific examples in the literature as needed. For the purpose of this study, social media, social media apps, and social networks are terms that are used interchangeably. In the initial screening, literature searches were conducted using combinations of keywords, such as “dermatology,” “dermatologists,” “dermatology journals,” “dermatology education,” “social media,” “social network,” “misinformation,” and “patient education.” A data analysis plan and inclusion and exclusion criteria were established before screening to minimize potential biases. A total of 1006 records were screened, of which, 895 were sought for retrieval and 768 were assessed for eligibility. Figure 1 shows the process using a PRISMA (Preferred Reporting Items for Systematic Reviews and Meta-Analyses) flow diagram. These sources were individually examined by the researcher for relevance and recency. Sources were included if their claims were corroborated in other peer-reviewed literature. Triangulation of sources was used to ensure that the data were valid and reliable. Each individually examined publication required a detailed review for inclusion to ensure relevance and ensure that the publication was focused on social media platforms and not just used in the discussion section. Study selection and subject area determinations were confirmed in consultation with a dermatology researcher with editorial experience in health care and social media. Disagreements were resolved in discussion with a third-party researcher in the department. Any duplicate results and non-English publications were excluded except for 1 article that had been translated into English from Spanish and another article that had been translated into English from German.

A narrative review format was selected as opposed to another type of systematic review because of its broader scope and ability to focus on observed trends. Narrative reviews typically do not require the presentation of reporting methodology, search terms, databases used, and inclusion and exclusion criteria; however, many of these are included in this study to ensure as much detail and transparency as possible [8]. The PRISMA guidelines checklist was adhered to complete this manuscript. For instance, these guidelines informed eligibility criteria for the studies, search strategy, certainty and bias assessments, implications for practice, and limitations [8]. The aim of this study was to formulate a narrative review of recent and relevant literature, so a qualitative analysis focused on examining the use of social media in dermatology with an emphasis on different social media platforms and different uses of social media. After the literature was evaluated, findings were synthesized, trends were established, and future implications were established. A small group of articles, grouped by subject areas established from observed trends, were selected for in-depth discussion.

## Results

### Overview

This narrative review draws from 42 recently published articles on patient education, social media usage, health misinformation, and dermatology. Many of these studies were published from research teams in the United States. However, a few of the studies were published in other countries, including Canada, Saudi Arabia, Turkey, Spain, Croatia, Germany, India, China, France, and Australia. These were analyzed after screening relevant literature, and many of the articles were published within the past 3 years. Of the current literature, there were several main subject areas including health misinformation, patient education, professionalism, and other potential uses of social media in dermatology. A narrative review of our findings has been outlined in the following sections, which have been organized based on these main subject areas. A summary of the studies used and examined, including country, year of publication, and platform, is summarized in Tables 1-4.

**Table 1:** Summary of articles in narrative review: health misinformation.

Authors	Country	Year of publication	Platform	Summary/key findings
Salah et al [9]	Saudi Arabia	2022	YouTube	Study shows a low overall accuracy and quality of YouTube videos on vitiligo.
Yeung et al [10]	Canada	2022	TikTok	Study shows approximately half of the analyzed TikTok videos about ADHD^a^ were misleading.
Szeto et al [4]	United States	2021	All	Study shows the potential for the propagation of inaccurate or even dangerous information is high.
Park et al [11]	United States	2018	Instagram	Study shows 45% of consumers report that social media health information influences their decision to seek care.
Yousaf et al [12]	United States	2020	Instagram, Reddit, YouTube	Study shows only 31% of participants consulting social media made changes fully aligned with AAD^b^ clinical guidelines.
Reddy [13]	United States	2021	Reddit	Study shows advice on the Reddit subreddit lacks evidence and pseudoscientific recommendations are often accepted as factual.
Zamil et al [14]	United States	2022	Instagram	Study shows that there is a prevalence of dermatologic supplements on social media with inaccurate health claims.

^a^ADHD: attention-deficit/hyperactivity disorder.

^b^AAD: American Academy of Dermatology.

**Table 2:** Summary of articles in narrative review: patient education.

Authors	Country	Year of publication	Platform	Summary/key findings
De Angelis et al [15]	Canada	2018	Discussion forums	Study shows health professionals find discussion forums and collaborative projects to be useful social media platforms to facilitate chronic disease self-management with patients.
Guzman et al [16]	United States	2020	YouTube	Study shows social media's merits to help facilitate chronic disease management for patients.
Cooper et al [17]	United States	2022	All	Study shows social media's use as a form of education for common dermatologic conditions.
Kaundinya et al [18]	United States	2020	All	Study shows that existing web-based resources for cirrhosis are too long and complex.
Karimkhani et al [19]	United States	2014	Instagram	Study shows how Instagram is used for engaging and informing patients.
Mansour et al [3]	United States	2022	TikTok	Study shows the effectiveness of TikTok patient education content for Keratosis Pilaris.
Nguyen et al [20]	United States	2021	TikTok	Study shows the content and creators making dermatology videos on TikTok.
Whitsitt et al [21]	United States	2015	Pinterest	Study shows informative pins were the most common (49%) followed by advocacy (37%) and home remedies (14%).
Daneshjou et al [22]	United States	2021	Twitter	Study shows how academic Twitter is used to spread health care information and promote research collaboration.
Chirumamilla and Gulati [23]	United States	2021	All	Study shows scientific communication on social media as a form of patient education benefits health literacy.
Boyers et al [24]	United States	2014	YouTube	Study shows of the total videos, 35% were uploaded by or featured an MD/DO/PhD in dermatology or other specialty/field, 2% FNP/PA, 1% RN, and 62% other.
Morrison et al [25]	United States	2019	Facebook	Study shows social media is an opportunity for targeted public health interventions for skin cancer.
Patel et al [26]	United States	2017	Snapchat	Study shows limited use of Snapchat by Dermatologists and professional entities.
Liakos et al [27]	United States	2021	Instagram	Study shows that social media, in particular Instagram, can be a successful platform to enhance the exposure of peer-reviewed medical information.
Correnti et al [28]	United States	2014	Tumblr	Study shows Tumblr remains a social media domain that lacks a strong presence from dermatology journals and organizations, remaining an untapped resource for information dissemination and interaction with the public.
Güder and Güder [29]	Turkey	2022	Instagram	Study shows hashtags used by physicians in their social media posts should be chosen from the words used in the folk language.
Taberner [30]	Spain	2015	All	Study shows that different social media sites may play different roles in patient education for dermatology.

**Table 3:** Summary of articles in narrative review: professionalism.

Authors	Country	Year of publication	Platform	Summary/key findings
Vukušić Rukavina et al [31]	Croatia	2021	All	Study shows existing recommendations for including e-professionalism in the educational curriculum for health care professionals.
Ahmed and Lipoff [6]	Germany	2022	All	Study shows benefits, risks, and role of dermatologists in social media content creation.
Janagond and Inamadar [32]	India	2021	WhatsApp	Study shows considerations for clinical photography utilizing WhatsApp.
Militello et al [33]	United States	2021	All	Study shows many dermatologists are currently influencers, but this requires formal training and the need to monitor online presence to prevent legal consequences.
Ko et al [7]	United States	2017	All	Study shows potential considerations for trainees and physician dermatological education.
Zhu et al [34]	China	2019	TikTok	Study shows the use of social media as a form of health communication for provincial health committees.

**Table 4:** Summary of articles in narrative review: other uses.

Authors	Country	Year of publication	Platform	Summary/key findings
Voillot et al [35]	France	2022	All	Study shows the benefit of social media support groups for patients with Atopic Dermatitis.
Hill et al [36]	United States	2018	Google+	Study shows the differential usage of Google+ for private practices in dermatology and dermatology journals.
Hopkins et al [37]	United States	2020	All	Study shows the influence of social media on cosmetic procedure popularity as measured by Google Trends.
Tan et al [38]	United States	2020	WeChat	Study shows the importance of cultural considerations in patient health-seeking behavior on social media.
Albeshri et al [5]	Saudi Arabia	2020	All	Study shows the role of social media in dermatologist selection by patients.
Patel et al [39]	United States	2018	All	Study shows among the professional dermatology organizations, 114 (47.1%) were on Facebook, 69 (28.5%) on Twitter, and 50 (20.7%) on LinkedIn. In comparison, 68 (87.2%) patient-centered organizations were on Facebook, 56 (71.8%) on Twitter, and 56 (71.8%) on LinkedIn.
Vasconcelos Silva et al [40]	Australia	2020	Twitter	Study shows sharing by celebrities or non-health–related organizations and individuals with a high following can all contribute to greater spread of skin cancer and sun-related messages.
St Claire et al [41]	United States	2019	All	Study shows of the 126 dermatology residency programs, 29 (23%) were active on Facebook, 14 (11%) on Twitter, and 9 (7%) on Instagram.
Petukhova et al [42]	United States	2020	Facebook	Study shows themes in a keratinocyte carcinoma support group including personal experience and provided psychosocial support (50%), there were a significant number of posts offering medical advice (35%), with most of such replies being unsupported by evidence-based medicine (87%)
Sharifzadeh and Smith [43]	United States	2022	Facebook, Twitter, and Instagram	Study shows that increased social media use resulted in a greater number of reviews, but not necessarily higher ratings.
Muralidhara and Paul [44]	United States	2018	Instagram	Study shows the most prevalent health information on social media is related to diet and exercise.
Gorman et al [45]	United States	2023	All	Studies have shown that skin cancer education should be expanded to include the skin of color patients and that social media is used by many individuals who otherwise do not have access to this information at frequent doctor appointments.
Zamil et al [14]	United States	2022	Instagram	Study shows that there is a prevalence of dermatologic supplements on social media with inaccurate health claims.

### Health Misinformation

Generally, it is known that health misinformation can spread quickly due to technology, such as social media platforms. On forums such as Reddit, which allow users to anonymously share information, a large portion of advice surrounding skincare lacks evidence, and pseudoscientific recommendations are also often accepted as factual [13]. This highlights one key issue with social media communities—the predisposition of these communities to becoming echo chambers that uphold pseudoscientific or otherwise not scientifically proven advice. To determine whether claims were valid, articles typically compared claims with American Academy of Dermatology (AAD) clinical guidelines for the condition and measured DISCERN scores. The latter, DISCERN, is a questionnaire that provides users with a valid and reliable way of assessing the quality of written information on treatment choices for a health issue [46]. Overall, health information on skincare for a variety of conditions appeared to contain misinformation across social media platforms.

However, misinformation may also be able to be combated through social media. A promising finding was that a recent study found that 68% of respondents who used social media for acne treatment advice were more likely to consult a medical professional [12]. This may result in more individuals seeking professional insight from dermatology professionals that may help counter misinformation encountered on social media. DISCERN scores indicate that there is a statistically significant difference in the quality of content between health care and non–health care sources (*P*=.009) [9]. Outside of dermatology content on social media, similar findings appear for other health information. Indeed, user-generated content on attention-deficit/hyperactivity disorder on TikTok has low actionability, with non–health care providers uploading the majority of misleading videos and health care providers uploading higher quality and more useful videos [10]. This may suggest that having more health care sources producing content can help increase the availability of quality health information on social media. On platforms like Instagram, self-identified dermatologists were responsible for only 16% of top dermatology posts, and only 5% of the top posts were made by the American Board of Dermatology–certified dermatologists [11]. This indicates that there are not only more posts made by nondermatologists on dermatology across social media but that more dermatologists using social media may provide higher-quality health information. Many of the articles agree that dermatologists should increase their presence on social media apps to counteract misleading information with evidence-based knowledge [4].

### Patient Education

A key component of this narrative review focuses on social media as a potential tool for patient education and distributing patient education materials. To determine the effectiveness of patient education materials, some studies used the Patient Education Materials Assessment Tool (Audiovisual Materials) to assess readability [18]. Considering that the average readability level in the United States is at a sixth-grade level or lower, many health materials may be perceived by individuals as difficult to understand [18]. It has been well established that young, White, and educated patients tend to have higher health literacy, whereas those from lower socioeconomic backgrounds, rural areas, and minority groups tend to have lower health literacy. Social media may provide an opportunity to close this health literacy gap by providing less complex patient education materials in a variety of formats, including video and infographics. Although social media is often associated with younger people, older demographics have also been increasingly using social media to increase their health literacy [23]. Patients are not only able to use social media to learn more about conditions but also use social media to lobby for decreases in disparities of care [23].

A main hurdle to understanding various health conditions has to do with the complex and often technical verbiage. A key part of the scientific process depends upon communication and dissemination of research findings, also referred to as scientific communication [22]. Many words typically used in academia and by physicians are often technical and, therefore, inaccessible. Simple changes such as using hashtags that use folk or colloquial language in lieu of complex terminology may help to improve accessibility [29]. Twitter, specifically a subset of users referred to as “academic Twitter,” provides a novel avenue for scientific communication. By using Twitter, the academic community can quickly disseminate information, and due to character limits on posts, much of this information is condensed and simplified [22]. A post, referred to on Twitter as a tweet, can consist of a single-sentence summary of a paper, an image of a key figure, the account names of the journal and scientists involved, and relevant keywords in hashtag format [22]. The use of social media to post articles demonstrated a significant (*P*<.0001) positive effect on both views (mean difference 175.5, SE 16.4) and downloads (mean difference 31.5, SE 4.0) when compared to matched articles not published on social media [27]. Social media may therefore be able to expand the reach of research articles and academic journals that are otherwise typically only accessed by individuals within the field.

Social media advertising, such as through Facebook Ads, is a feasible approach to reaching individuals within a target population with public health interventions [25]. Indeed, Facebook Ads allow users to specify a target demographic including their age, location, and other pieces of information. Compared to traditional outlets, health behavior changes, such as avoiding tanning beds to prevent skin cancer, can be quickly brought to the attention of relevant users [25]. Across the major social media sites such as Instagram, Pinterest, YouTube, Snapchat, and Tumblr, as recently as 2015 many journals and private practices did not have accounts [19,21,24,26,28]. This means the use of targeted social media advertising and, more broadly, the usage of social media by the dermatology community is a very recent phenomenon. TikTok, the largest growing social media network since 2019, has rapidly become one of the most used social media platforms for accessing health posts [20]. TikTok is also perhaps one of the most user-catered social media networks in use today. A key feature of TikTok is the For You Page that provides each user with tailored video content based on their interests, likes, and dislikes. As patients increasingly turn to social media for health information, dermatology-related TikTok videos have gained appeal as they provide education by laypeople for laypeople [20]. Yet, unlike other sites, board-certified dermatologists accounted for 15.1% of total posts but authored a significant percentage of posts with the hashtag “dermatology” (45%) [20]. This may suggest a higher presence of dermatologists on TikTok. Regardless, it will be important for dermatologists to continue to adapt to digital media to find new and efficient ways to communicate with patients and the broader scientific community [30].

### Professionalism

There exist several benefits and dangers of social media not only on patient education but also professionalism on the part of dermatologists and trainees. Some dangers of social media on professionalism are as follows: (1) less accountability, (2) breaching confidentiality, (3) blurred professional boundaries, (4) depiction of unprofessional behavior, and (5) legal issues [31]. These dangers may also exist when some health professionals use social media platforms to facilitate disease self-management, although this is still a relatively rare phenomenon due to the amount of time required by the physician [15]. However, a notable benefit to social media usage is that each platform has unique qualities and features that people can use to educate and learn [17]. Currently, the top 5 platforms are Twitter, Instagram, TikTok, YouTube, and Facebook [17]. So long as providers, such as dermatologists, take ethical considerations into account these platforms can provide patients with educational content catered to their learning style and preferred modality [17]. Dermatologists must also balance creating educational content and maintaining ethical standards while also remaining transparent about commercial interests such as brand sponsorships [16,33].

### Other Potential Uses of Social Media in Dermatology

It is important to consider how social media provides users with support and knowledge which can help them better advocate for themselves in health care settings. Support groups on social media can be a form of psychosocial support since these groups allow individuals to share their personal experiences and openly discuss their concerns [42]. Private groups, such as those on Facebook for patients with skin cancer, can allow for a close-knit community experience on a large social networking site [42]. Because patients are able to candidly discuss treatments, their perceptions, and commentary on their quality of life, social media can be a crucial tool in the development of new approaches that consider patient concerns [35]. Beyond psychosocial support, patients can use these forums to broaden their understanding of their condition and treatment options, which can empower them to have more productive discussions with their health care provider and to ultimately make more informed health decisions.

Another potential use of social media in dermatology is the promotion and marketing of academic journals and trainee programs. However, social media has been underutilized in this space. Google+ is another example of a social media site and its uniqueness lies in its search engine optimization services. Although some private practices are on Google+, the majority of dermatology journals have yet to use Google+ to expand their audiences [36]. As of 2018, there were 22 (17.7%) dermatology journals active on Facebook and 21 (16.9%) on Twitter [39]. Among the professional dermatology organizations, 114 (47.1%) were on Facebook, 69 (28.5%) on Twitter, and 50 (20.7%) on LinkedIn [39]. This suggests that dermatology journals have been slower at using social media and establishing a social media presence compared to professional organizations and private practices. Residency programs for dermatology have had similarly slow social media uptake. As of 2019, of the 126 dermatology residency programs, 29 (23%) were active on Facebook, 14 (11%) on Twitter, and 9 (7%) on Instagram [41]. Other groups, such as Provincial Health Committees in China, have started to expand their TikTok presence to engage with local residents and communicate public health information [34].

Yet another use of social media in dermatology is to promote private practices and individual providers. However, it is crucial to reiterate that dermatologists must do so while maintaining ethical standards, remaining transparent about commercial interests such as brand sponsorships, and maintaining patient consent for sharing images [16,32,33]. Not all social media sites are created equal. Interestingly, the terms *dermatologist, Botox, Juvederm, Radiesse, CoolSculpting*, and *Kybella* were associated with both Instagram and Facebook users, but *blepharoplasty* and *rhinoplasty* were only associated with Instagram users (*P*<.01) [37]. Although many individuals state that they trust Dermatologists, celebrities with a high number of followers also achieve a substantial amount of likes and influence on skin cancer–related communication on sites like Twitter [40]. Besides sharing educational content, dermatologists should also remain up-to-date on understanding social media trends and advising patients to exercise caution in regard to invasive, potentially dangerous cosmetic procedures without doing their own research [38,44]. With these considerations in mind, many Dermatologists are using social media to promote themselves and their private practices. Many patients learn about their dermatologist from social media and dermatologists who maintain a social media presence typically have higher web-based reviews [5,43]. All this is to say, there are benefits for dermatologists to stay up-to-date on social media.

## Discussion

### Principal Results

It can be suggested that social media has been leveraged as the latest form of patient education in dermatology. Using social media can help to bridge patient-provider relationships, provide social support, enhance patient understanding of treatment options, spread current research information previously only shared in academic spheres, and cater content based on user preferences. This is a novel approach to scientific communication, health literacy, and patient education and will help to bridge disparities that lead to subpar health care in dermatology. Although social media’s ability to provide catered user-centered content has yielded favorable results, challenges persist regarding efficient and appropriate use of social media as a form of patient education. Therefore, dermatologists and trainees must consider pitfalls to social media as a form of patient education, such as less accountability, blurred professional boundaries, and challenges in maintaining transparency about commercial interests [16,31]. Despite this, dermatologists that use social media have reaped immediate benefits, including a broader client base and higher web-based reviews for their private practices [5,43]. For patients, social media is not only useful as an overt form of patient education but also a means to find community, support and ultimately have the tools to make informed health care decisions [42]. Some limitations of this narrative review include the exclusion of publications that are not indexed in PubMed. Future reviews should build upon this initial survey to assess a broader scope of literature and potentially establish other potential uses of social media in dermatology.

It is important to consider that innovations in technology will continue to increase the number of people turning to social media and telemedicine for health information, but that tailored content can also have legal implications. To develop patient education content on social media, it is important to consider the different demographics that access various social media sites as demonstrated [1]. For instance, 37% of social media news consumers are Republican or lean Republican [1]. Education level varies across platforms. Facebook, Instagram, and TikTok had 28%, 30%, and 15% of users with a college degree or higher, respectively [1]. Using this information can help with content development that is catered to the users’ demographic information. There is a great realm of potential for providers and patients to become more informed on treatment options and common concerns to improve bidirectional communication in health care settings [42]. Although catered, easily accessible content is part of the appeal of social media as an avenue for patient education, its use may also trigger legal implications because of not only blurred professional boundaries, but rising concerns over data privacy and social media [31,47].

Although tailored content is a promising avenue for public health interventions and preventative health care in dermatology, targeted messaging can be applied to affect patient behavior, bypassing existing regulations on disclosure and informed consent and thus raising legal implications [14,45,47]. This shows the need for subsequent research on these implications and potential solutions to deliver patient education content without bypassing existing regulations and ethical guidelines.

### Implications

Perhaps most promising is the potential of the dermatology community to directly combat health misinformation through social media. The use of social media to post research has demonstrated a significant (*P*<.0001) positive effect on both views (mean difference 175.5, SE 16.4) and downloads (mean difference 31.5, SE 4.0) when compared to matched articles not published on social media [27]. This suggests social media play an important role in the spread of the most recent innovations. As peer-reviewed research becomes more available and accessible, patients and social media users will be able to make more informed health care decisions. Some social media platforms are already seeing increases in content created by board-certified dermatologists such as on TikTok where board-certified dermatologists accounted for 15.1% of total posts but authored a significant percentage of posts with the hashtag “dermatology” (45%) [20]. Unique features of different social media sites can allow for additional moderation and curb the spread of medical misinformation. TikTok’s Duet feature allows physicians and scientists to directly refute medical misinformation, Facebook has instituted a Coordinated Harm Policy (and related policies) that removes misinformation content, and Twitter now contains a disclaimer that urges users to read articles before sharing content.

### Limitations

There are a few limitations of this current narrative review. There is not yet an established set of guidelines for validating health information on social media which are applicable to all the different platforms. Therefore, this review is unable to establish a generalizable set of best practices for tackling this concern. The narrative review methodology was used, which provided advantages such as a more inclusive picture of available research and the ability to provide rationales for future research. However, the weaknesses of the narrative review methodology include selection bias. To limit, though not entirely eliminate bias, the author established explicit criteria for article selection and used multiple databases. This review did not have other full-time contributors although other experts were consulted during the initial stages to reduce the risk of bias. Another limitation is that this narrative review does not include differences in behavior across intersectional demographic groups. Some of the selected papers may have their own biases as some may be written by groups or individuals who currently use social media for patient education or health information. Many of the studies in this review are from the United States and most studies in this review are from predominantly White, educated, industrialized, rich, and democratic societies (WEIRD societies). However, social media is used globally, and several studies analyzed global trends and data from social media regardless of the country of origin of the researchers.

### Conclusions

Social media has rapidly become a staple in the daily lives of billions. Social media has become a modality for patient education, misinformation, and marketing in dermatology. Although there are many unique features across all platforms that allow for the rapid and simplified dissemination of research and relevant health information, misinformation spreads equally as well. Integration of social media into dermatology practice has risen in prevalence in the past few years, but there continue to be avenues to expand the social media presence of academic Dermatology and private practices alike. Examining successful social media strategies while ensuring ethical standards of dermatology is upheld and will be crucial to the continued growth of social media as a form of dermatologic patient education. This narrative review aims to highlight current uses of social media, pitfalls, and benefits of social media use for patient education and emerging applications for social media usage in dermatology. Further research should aim to analyze the efficacy of different strategies to mitigate health misinformation on social media. Future studies and innovations should also continue to explore innovations in this space, efficacy of different modalities of posts, and longitudinal differences in patient outcomes and health literacy. It is especially important for future studies to explore whether existing social media patient education materials provide culturally competent and diverse representations.

## Abbreviations

AAD: Academy of Dermatology
ADHD: attention-deficit/hyperactivity disorder
PRISMA: Preferred Reporting Items for Systematic Reviews and Meta-Analyses
